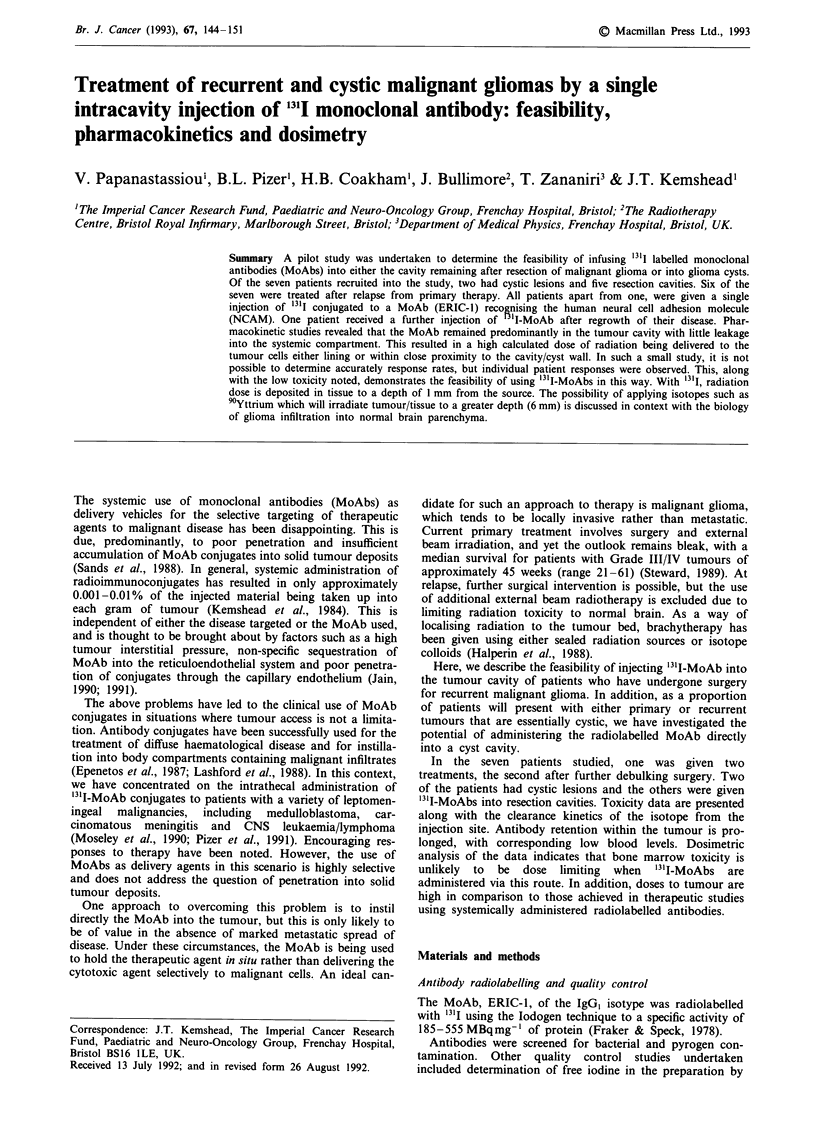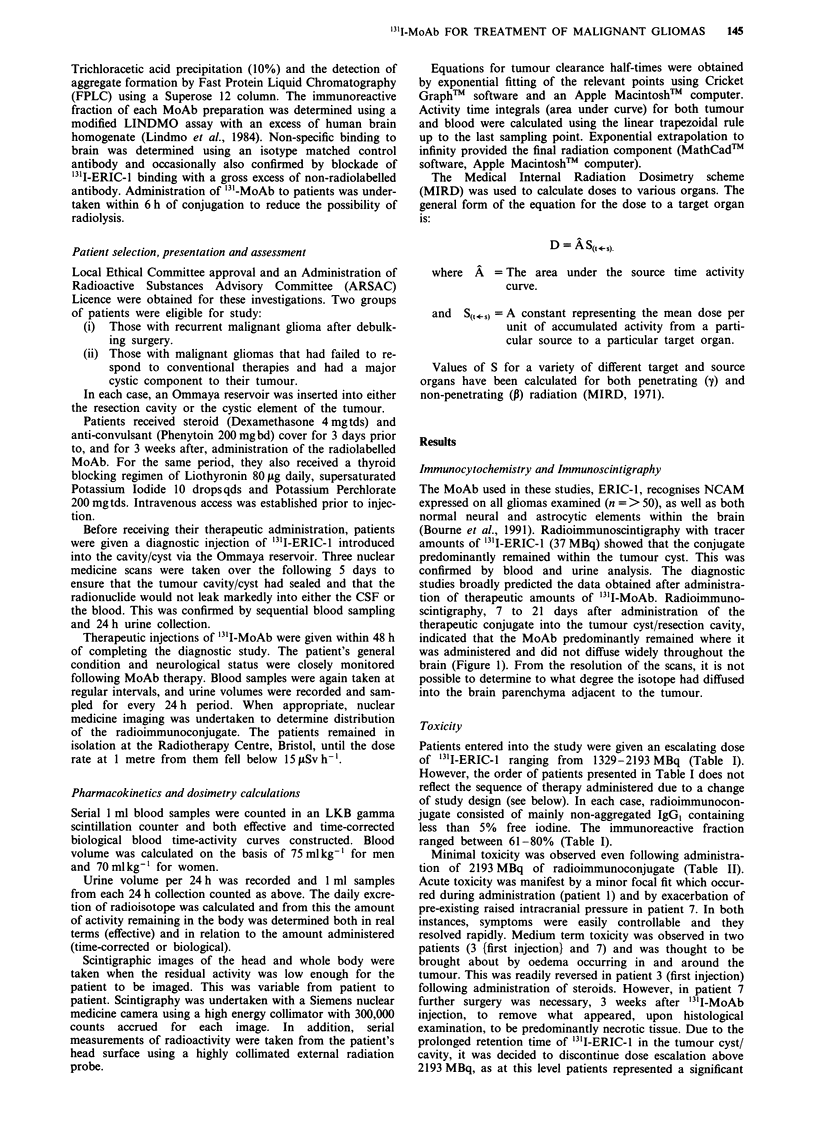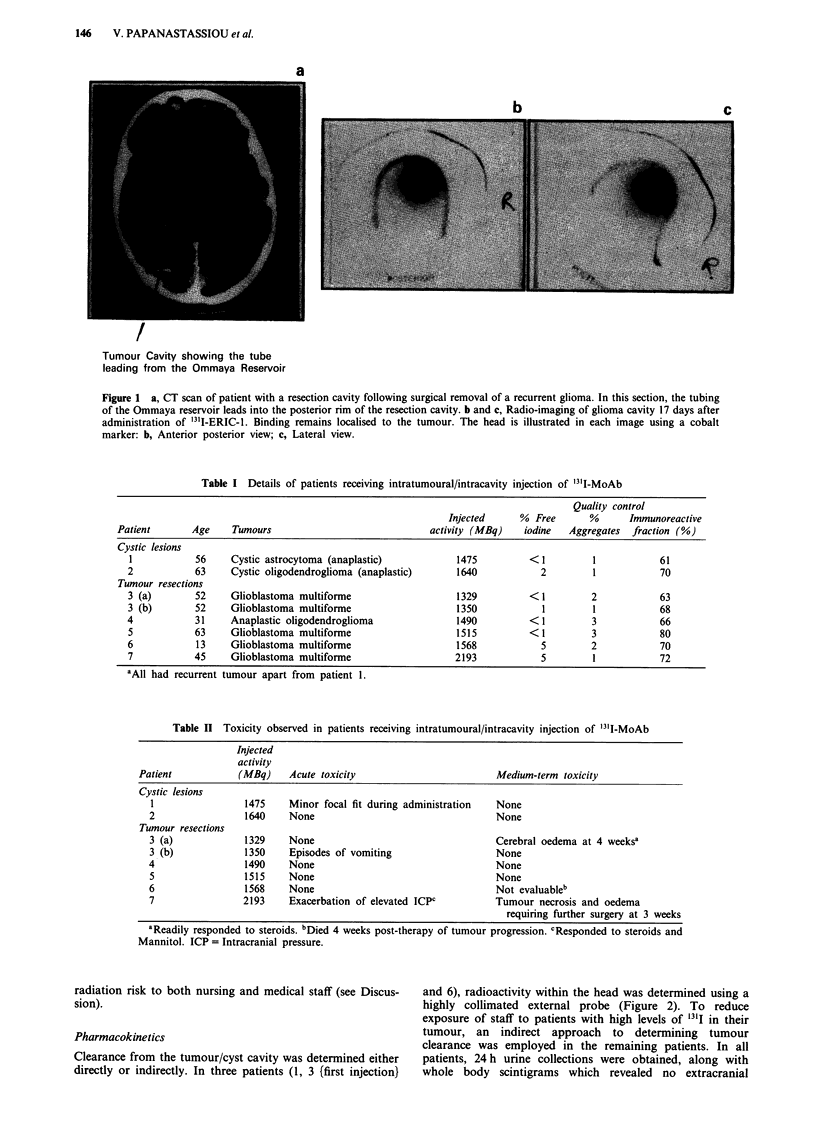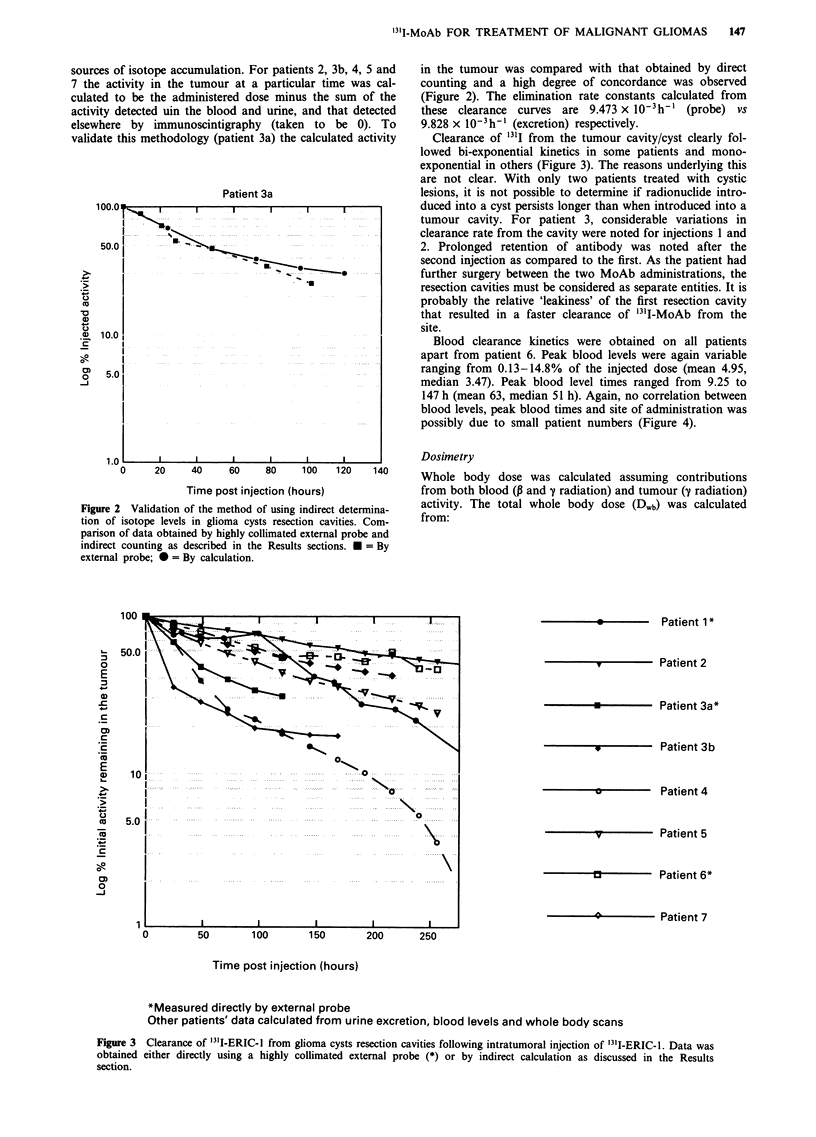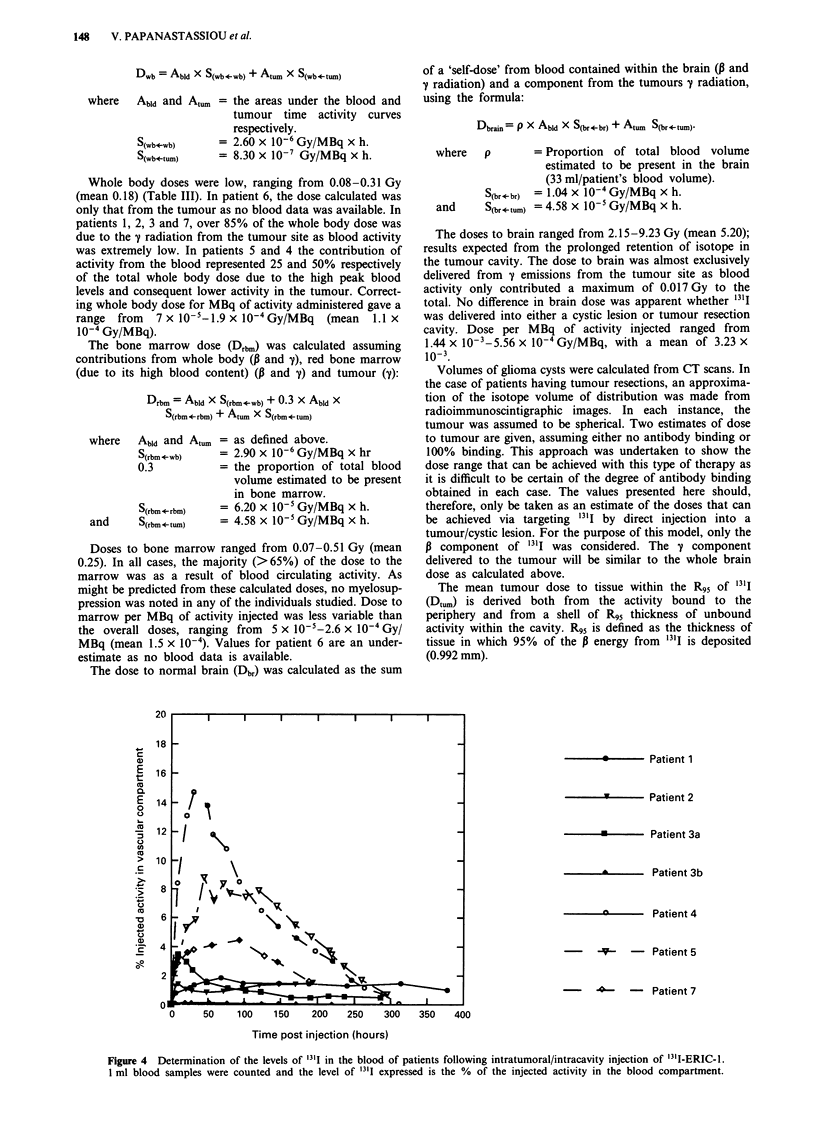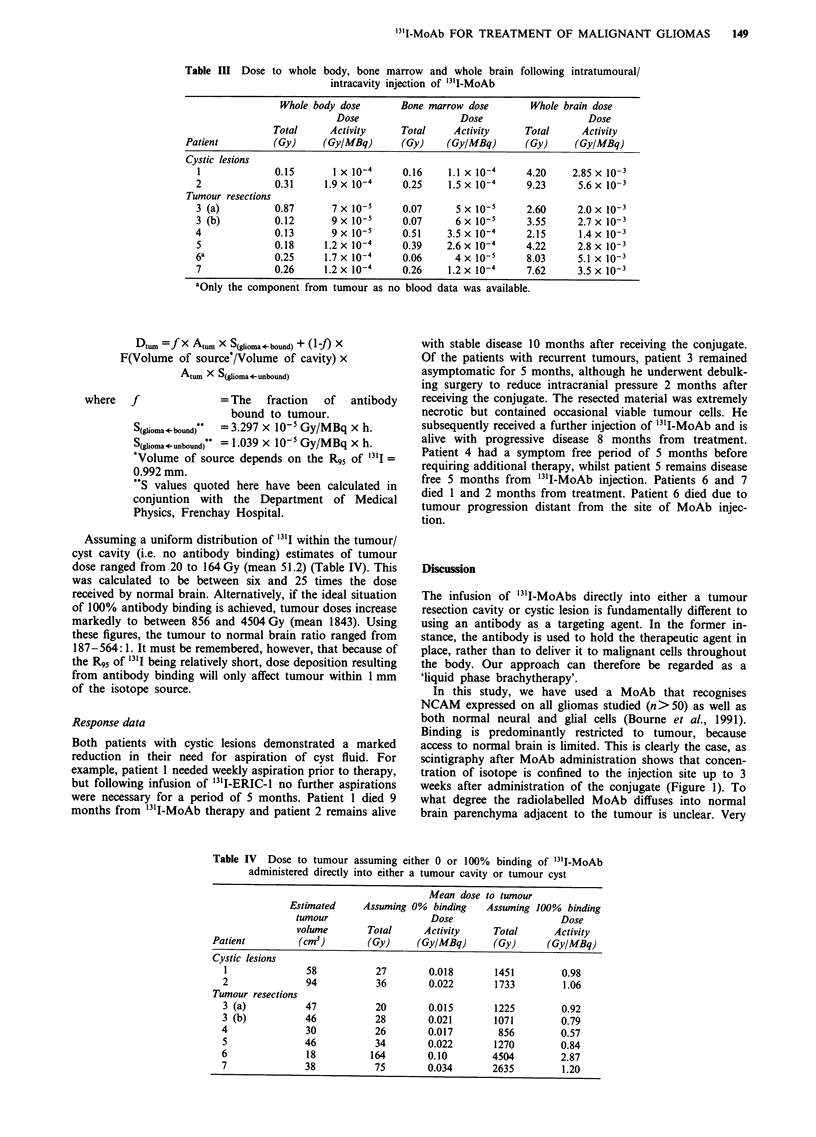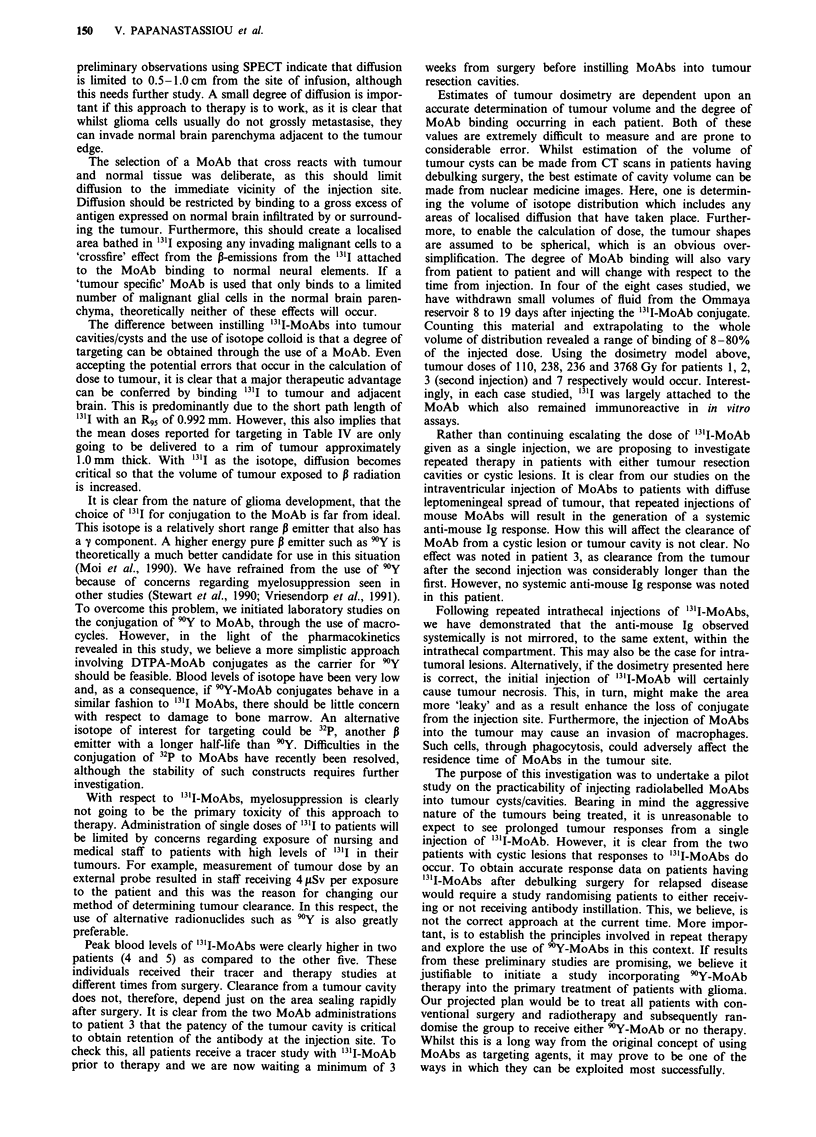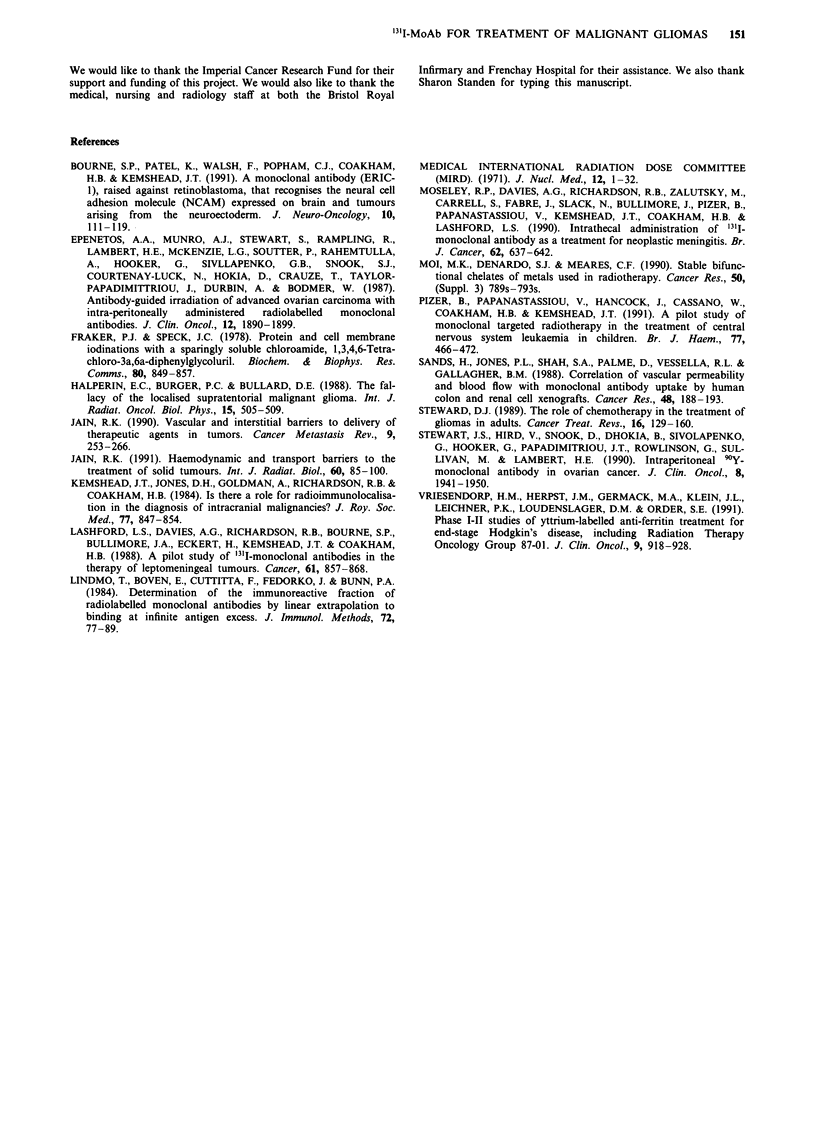# Treatment of recurrent and cystic malignant gliomas by a single intracavity injection of 131I monoclonal antibody: feasibility, pharmacokinetics and dosimetry.

**DOI:** 10.1038/bjc.1993.25

**Published:** 1993-01

**Authors:** V. Papanastassiou, B. L. Pizer, H. B. Coakham, J. Bullimore, T. Zananiri, J. T. Kemshead

**Affiliations:** Imperial Cancer Research Fund, Frenchay Hospital, Bristol, UK.

## Abstract

**Images:**


Br. J. Cancer (1993), 67, 144–151 © Macmillan Press Ltd., 1993

### Treatment of recurrent and cystic malignant gliomas by a single intracavity injection of $^{131}$I monoclonal antibody: feasibility, pharmacokinetics and dosimetry

V. Papanastassiou[1], B.L. Pizer[1], H.B. Coakham[1], J. Bullimore[2], T. Zananiri[3] & J.T. Kemshead[1]

[1]*The Imperial Cancer Research Fund, Paediatric and Neuro-Oncology Group, Frenchay Hospital, Bristol;* [2]*The Radiotherapy Centre, Bristol Royal Infirmary, Marlborough Street, Bristol;* [3]*Department of Medical Physics, Frenchay Hospital, Bristol, UK.*

**Summary** A pilot study was undertaken to determine the feasibility of infusing $^{131}$I labelled monoclonal antibodies (MoAbs) into either the cavity remaining after resection of malignant glioma or into glioma cysts. Of the seven patients recruited into the study, two had cystic lesions and five resection cavities. Six of the seven were treated after relapse from primary therapy. All patients apart from one, were given a single injection of $^{131}$I conjugated to a MoAb (ERIC-1) recognising the human neural cell adhesion molecule (NCAM). One patient received a further injection of $^{131}$I-MoAb after regrowth of their disease. Pharmacokinetic studies revealed that the MoAb remained predominantly in the tumour cavity with little leakage into the systemic compartment. This resulted in a high calculated dose of radiation being delivered to the tumour cells either lining or within close proximity to the cavity/cyst wall. In such a small study, it is not possible to determine accurately response rates, but individual patient responses were observed. This, along with the low toxicity noted, demonstrates the feasibility of using $^{131}$I-MoAbs in this way. With $^{131}$I, radiation dose is deposited in tissue to a depth of 1 mm from the source. The possibility of applying isotopes such as $^{90}$Yttrium which will irradiate tumour/tissue to a greater depth (6 mm) is discussed in context with the biology of glioma infiltration into normal brain parenchyma.

The systemic use of monoclonal antibodies (MoAbs) as delivery vehicles for the selective targeting of therapeutic agents to malignant disease has been disappointing. This is due, predominantly, to poor penetration and insufficient accumulation of MoAb conjugates into solid tumour deposits (Sands *et al.*, 1988). In general, systemic administration of radioimmunoconjugates has resulted in only approximately 0.001–0.01% of the injected material being taken up into each gram of tumour (Kemshead *et al.*, 1984). This is independent of either the disease targeted or the MoAb used, and is thought to be brought about by factors such as a high tumour interstitial pressure, non-specific sequestration of MoAb into the reticuloendothelial system and poor penetration of conjugates through the capillary endothelium (Jain, 1990; 1991).

The above problems have led to the clinical use of MoAb conjugates in situations where tumour access is not a limitation. Antibody conjugates have been successfully used for the treatment of diffuse haematological disease and for instillation into body compartments containing malignant infiltrates (Epenetos *et al.*, 1987; Lashford *et al.*, 1988). In this context, we have concentrated on the intrathecal administration of $^{131}$I-MoAb conjugates to patients with a variety of leptomeningeal malignancies, including medulloblastoma, carcinomatous meningitis and CNS leukaemia/lymphoma (Moseley *et al.*, 1990; Pizer *et al.*, 1991). Encouraging responses to therapy have been noted. However, the use of MoAbs as delivery agents in this scenario is highly selective and does not address the question of penetration into solid tumour deposits.

One approach to overcoming this problem is to instil directly the MoAb into the tumour, but this is only likely to be of value in the absence of marked metastatic spread of disease. Under these circumstances, the MoAb is being used to hold the therapeutic agent *in situ* rather than delivering the cytotoxic agent selectively to malignant cells. An ideal candidate for such an approach to therapy is malignant glioma, which tends to be locally invasive rather than metastatic. Current primary treatment involves surgery and external beam irradiation, and yet the outlook remains bleak, with a median survival for patients with Grade III/IV tumours of approximately 45 weeks (range 21–61) (Steward, 1989). At relapse, further surgical intervention is possible, but the use of additional external beam radiotherapy is excluded due to limiting radiation toxicity to normal brain. As a way of localising radiation to the tumour bed, brachytherapy has been given using either sealed radiation sources or isotope colloids (Halperin *et al.*, 1988).

Here, we describe the feasibility of injecting $^{131}$I-MoAb into the tumour cavity of patients who have undergone surgery for recurrent malignant glioma. In addition, as a proportion of patients will present with either primary or recurrent tumours that are essentially cystic, we have investigated the potential of administering the radiolabelled MoAb directly into a cyst cavity.

In the seven patients studied, one was given two treatments, the second after further debulking surgery. Two of the patients had cystic lesions and the others were given $^{131}$I-MoAbs into resection cavities. Toxicity data are presented along with the clearance kinetics of the isotope from the injection site. Antibody retention within the tumour is prolonged, with corresponding low blood levels. Dosimetric analysis of the data indicates that bone marrow toxicity is unlikely to be dose limiting when $^{131}$I-MoAbs are administered via this route. In addition, doses to tumour are high in comparison to those achieved in therapeutic studies using systemically administered radiolabelled antibodies.

### Materials and methods

### *Antibody radiolabelling and quality control*

The MoAb, ERIC-1, of the $IgG_1$ isotype was radiolabelled with $^{131}$I using the Iodogen technique to a specific activity of 185–555 MBq mg$^{-1}$ of protein (Fraker & Speck, 1978).

Antibodies were screened for bacterial and pyrogen contamination. Other quality control studies undertaken included determination of free iodine in the preparation by

Correspondence: J.T. Kemshead, The Imperial Cancer Research Fund, Paediatric and Neuro-Oncology Group, Frenchay Hospital, Bristol BS16 1LE, UK.
Received 13 July 1992; and in revised form 26 August 1992.

Trichloracetic acid precipitation (10%) and the detection of aggregate formation by Fast Protein Liquid Chromatography (FPLC) using a Superose 12 column. The immunoreactive fraction of each MoAb preparation was determined using a modified LINDMO assay with an excess of human brain homogenate (Lindmo *et al.*, 1984). Non-specific binding to brain was determined using an isotype matched control antibody and occasionally also confirmed by blockade of $^{131}$I-ERIC-1 binding with a gross excess of non-radiolabelled antibody. Administration of $^{131}$-MoAb to patients was undertaken within 6 h of conjugation to reduce the possibility of radiolysis.

*Patient selection, presentation and assessment*

Local Ethical Committee approval and an Administration of Radioactive Substances Advisory Committee (ARSAC) Licence were obtained for these investigations. Two groups of patients were eligible for study:

(i) Those with recurrent malignant glioma after debulking surgery.
(ii) Those with malignant gliomas that had failed to respond to conventional therapies and had a major cystic component to their tumour.

In each case, an Ommaya reservoir was inserted into either the resection cavity or the cystic element of the tumour.

Patients received steroid (Dexamethasone 4 mg tds) and anti-convulsant (Phenytoin 200 mg bd) cover for 3 days prior to, and for 3 weeks after, administration of the radiolabelled MoAb. For the same period, they also received a thyroid blocking regimen of Liothyronin 80 μg daily, supersaturated Potassium Iodide 10 drops qds and Potassium Perchlorate 200 mg tds. Intravenous access was established prior to injection.

Before receiving their therapeutic administration, patients were given a diagnostic injection of $^{131}$I-ERIC-1 introduced into the cavity/cyst via the Ommaya reservoir. Three nuclear medicine scans were taken over the following 5 days to ensure that the tumour cavity/cyst had sealed and that the radionuclide would not leak markedly into either the CSF or the blood. This was confirmed by sequential blood sampling and 24 h urine collection.

Therapeutic injections of $^{131}$I-MoAb were given within 48 h of completing the diagnostic study. The patient's general condition and neurological status were closely monitored following MoAb therapy. Blood samples were again taken at regular intervals, and urine volumes were recorded and sampled for every 24 h period. When appropriate, nuclear medicine imaging was undertaken to determine distribution of the radioimmunoconjugate. The patients remained in isolation at the Radiotherapy Centre, Bristol, until the dose rate at 1 metre from them fell below 15 $\mu Sv\,h^{-1}$.

*Pharmacokinetics and dosimetry calculations*

Serial 1 ml blood samples were counted in an LKB gamma scintillation counter and both effective and time-corrected biological blood time-activity curves constructed. Blood volume was calculated on the basis of 75 $ml\,kg^{-1}$ for men and 70 $ml\,kg^{-1}$ for women.

Urine volume per 24 h was recorded and 1 ml samples from each 24 h collection counted as above. The daily excretion of radioisotope was calculated and from this the amount of activity remaining in the body was determined both in real terms (effective) and in relation to the amount administered (time-corrected or biological).

Scintigraphic images of the head and whole body were taken when the residual activity was low enough for the patient to be imaged. This was variable from patient to patient. Scintigraphy was undertaken with a Siemens nuclear medicine camera using a high energy collimator with 300,000 counts accrued for each image. In addition, serial measurements of radioactivity were taken from the patient's head surface using a highly collimated external radiation probe.

Equations for tumour clearance half-times were obtained by exponential fitting of the relevant points using Cricket Graph™ software and an Apple Macintosh™ computer. Activity time integrals (area under curve) for both tumour and blood were calculated using the linear trapezoidal rule up to the last sampling point. Exponential extrapolation to infinity provided the final radiation component (MathCad™ software, Apple Macintosh™ computer).

The Medical Internal Radiation Dosimetry scheme (MIRD) was used to calculate doses to various organs. The general form of the equation for the dose to a target organ is:

$$D = \hat{A}\,S_{(t \leftarrow s)}.$$

where $\hat{A}$ = The area under the source time activity curve.

and $S_{(t \leftarrow s)}$ = A constant representing the mean dose per unit of accumulated activity from a particular source to a particular target organ.

Values of S for a variety of different target and source organs have been calculated for both penetrating (γ) and non-penetrating (β) radiation (MIRD, 1971).

### Results

*Immunocytochemistry and Immunoscintigraphy*

The MoAb used in these studies, ERIC-1, recognises NCAM expressed on all gliomas examined ($n = > 50$), as well as both normal neural and astrocytic elements within the brain (Bourne *et al.*, 1991). Radioimmunoscintigraphy with tracer amounts of $^{131}$I-ERIC-1 (37 MBq) showed that the conjugate predominantly remained within the tumour cyst. This was confirmed by blood and urine analysis. The diagnostic studies broadly predicted the data obtained after administration of therapeutic amounts of $^{131}$I-MoAb. Radioimmunoscintigraphy, 7 to 21 days after administration of the therapeutic conjugate into the tumour cyst/resection cavity, indicated that the MoAb predominantly remained where it was administered and did not diffuse widely throughout the brain (Figure 1). From the resolution of the scans, it is not possible to determine to what degree the isotope had diffused into the brain parenchyma adjacent to the tumour.

*Toxicity*

Patients entered into the study were given an escalating dose of $^{131}$I-ERIC-1 ranging from 1329–2193 MBq (Table I). However, the order of patients presented in Table I does not reflect the sequence of therapy administered due to a change of study design (see below). In each case, radioimmunoconjugate consisted of mainly non-aggregated $IgG_1$ containing less than 5% free iodine. The immunoreactive fraction ranged between 61–80% (Table I).

Minimal toxicity was observed even following administration of 2193 MBq of radioimmunoconjugate (Table II). Acute toxicity was manifest by a minor focal fit which occurred during administration (patient 1) and by exacerbation of pre-existing raised intracranial pressure in patient 7. In both instances, symptoms were easily controllable and they resolved rapidly. Medium term toxicity was observed in two patients (3 {first injection} and 7) and was thought to be brought about by oedema occurring in and around the tumour. This was readily reversed in patient 3 (first injection) following administration of steroids. However, in patient 7 further surgery was necessary, 3 weeks after $^{131}$I-MoAb injection, to remove what appeared, upon histological examination, to be predominantly necrotic tissue. Due to the prolonged retention time of $^{131}$I-ERIC-1 in the tumour cyst/cavity, it was decided to discontinue dose escalation above 2193 MBq, as at this level patients represented a significant

**Figure 1** **a**, CT scan of patient with a resection cavity following surgical removal of a recurrent glioma. In this section, the tubing of the Ommaya reservoir leads into the posterior rim of the resection cavity. **b** and **c**, Radio-imaging of glioma cavity 17 days after administration of $^{131}$I-ERIC-1. Binding remains localised to the tumour. The head is illustrated in each image using a cobalt marker: **b**, Anterior posterior view; **c**, Lateral view.

**Table I** Details of patients receiving intratumoural/intracavity injection of $^{131}$I-MoAb

| *Patient* | *Age* | *Tumours* | *Injected activity (MBq)* | *% Free iodine* | *Quality control % Aggregates* | *Immunoreactive fraction (%)* |
|---|---|---|---|---|---|---|
| *Cystic lesions* | | | | | | |
| 1 | 56 | Cystic astrocytoma (anaplastic) | 1475 | <1 | 1 | 61 |
| 2 | 63 | Cystic oligodendroglioma (anaplastic) | 1640 | 2 | 1 | 70 |
| *Tumour resections* | | | | | | |
| 3 (a) | 52 | Glioblastoma multiforme | 1329 | <1 | 2 | 63 |
| 3 (b) | 52 | Glioblastoma multiforme | 1350 | 1 | 1 | 68 |
| 4 | 31 | Anaplastic oligodendroglioma | 1490 | <1 | 3 | 66 |
| 5 | 63 | Glioblastoma multiforme | 1515 | <1 | 3 | 80 |
| 6 | 13 | Glioblastoma multiforme | 1568 | 5 | 2 | 70 |
| 7 | 45 | Glioblastoma multiforme | 2193 | 5 | 1 | 72 |

[a]All had recurrent tumour apart from patient 1.

**Table II** Toxicity observed in patients receiving intratumoural/intracavity injection of $^{131}$I-MoAb

| *Patient* | *Injected activity (MBq)* | *Acute toxicity* | *Medium-term toxicity* |
|---|---|---|---|
| *Cystic lesions* | | | |
| 1 | 1475 | Minor focal fit during administration | None |
| 2 | 1640 | None | None |
| *Tumour resections* | | | |
| 3 (a) | 1329 | None | Cerebral oedema at 4 weeks[a] |
| 3 (b) | 1350 | Episodes of vomiting | None |
| 4 | 1490 | None | None |
| 5 | 1515 | None | None |
| 6 | 1568 | None | Not evaluable[b] |
| 7 | 2193 | Exacerbation of elevated ICP[c] | Tumour necrosis and oedema requiring further surgery at 3 weeks |

[a]Readily responded to steroids. [b]Died 4 weeks post-therapy of tumour progression. [c]Responded to steroids and Mannitol. ICP = Intracranial pressure.

radiation risk to both nursing and medical staff (see Discussion).

### *Pharmacokinetics*

Clearance from the tumour/cyst cavity was determined either directly or indirectly. In three patients (1, 3 {first injection} and 6), radioactivity within the head was determined using a highly collimated external probe (Figure 2). To reduce exposure of staff to patients with high levels of $^{131}$I in their tumour, an indirect approach to determining tumour clearance was employed in the remaining patients. In all patients, 24 h urine collections were obtained, along with whole body scintigrams which revealed no extracranial sources of isotope accumulation. For patients 2, 3b, 4, 5 and 7 the activity in the tumour at a particular time was calculated to be the administered dose minus the sum of the activity detected uin the blood and urine, and that detected elsewhere by immunoscintigraphy (taken to be 0). To validate this methodology (patient 3a) the calculated activity in the tumour was compared with that obtained by direct counting and a high degree of concordance was observed (Figure 2). The elimination rate constants calculated from these clearance curves are $9.473 \times 10^{-3}h^{-1}$ (probe) *vs* $9.828 \times 10^{-3}h^{-1}$ (excretion) respectively.

**Figure 2** Validation of the method of using indirect determination of isotope levels in glioma cysts resection cavities. Comparison of data obtained by highly collimated external probe and indirect counting as described in the Results sections. ■ = By external probe; ● = By calculation.

Clearance of $^{131}$I from the tumour cavity/cyst clearly followed bi-exponential kinetics in some patients and mono-exponential in others (Figure 3). The reasons underlying this are not clear. With only two patients treated with cystic lesions, it is not possible to determine if radionuclide introduced into a cyst persists longer than when introduced into a tumour cavity. For patient 3, considerable variations in clearance rate from the cavity were noted for injections 1 and 2. Prolonged retention of antibody was noted after the second injection as compared to the first. As the patient had further surgery between the two MoAb administrations, the resection cavities must be considered as separate entities. It is probably the relative 'leakiness' of the first resection cavity that resulted in a faster clearance of $^{131}$I-MoAb from the site.

Blood clearance kinetics were obtained on all patients apart from patient 6. Peak blood levels were again variable ranging from 0.13–14.8% of the injected dose (mean 4.95, median 3.47). Peak blood level times ranged from 9.25 to 147 h (mean 63, median 51 h). Again, no correlation between blood levels, peak blood times and site of administration was possibly due to small patient numbers (Figure 4).

*Dosimetry*

Whole body dose was calculated assuming contributions from both blood ($\beta$ and $\gamma$ radiation) and tumour ($\gamma$ radiation) activity. The total whole body dose ($D_{wb}$) was calculated from:

**Figure 3** Clearance of $^{131}$I-ERIC-1 from glioma cysts resection cavities following intratumoral injection of $^{131}$I-ERIC-1. Data was obtained either directly using a highly collimated external probe (*) or by indirect calculation as discussed in the Results section.

$$D_{wb} = A_{bld} \times S_{(wb \leftarrow wb)} + A_{tum} \times S_{(wb \leftarrow tum)}$$

where $A_{bld}$ and $A_{tum}$ = the areas under the blood and tumour time activity curves respectively.

$S_{(wb \leftarrow wb)}$ = $2.60 \times 10^{-6}$ Gy/MBq × h.

$S_{(wb \leftarrow tum)}$ = $8.30 \times 10^{-7}$ Gy/MBq × h.

Whole body doses were low, ranging from 0.08–0.31 Gy (mean 0.18) (Table III). In patient 6, the dose calculated was only that from the tumour as no blood data was available. In patients 1, 2, 3 and 7, over 85% of the whole body dose was due to the $\gamma$ radiation from the tumour site as blood activity was extremely low. In patients 5 and 4 the contribution of activity from the blood represented 25 and 50% respectively of the total whole body dose due to the high peak blood levels and consequent lower activity in the tumour. Correcting whole body dose for MBq of activity administered gave a range from $7 \times 10^{-5}$–$1.9 \times 10^{-4}$ Gy/MBq (mean $1.1 \times 10^{-4}$ Gy/MBq).

The bone marrow dose ($D_{rbm}$) was calculated assuming contributions from whole body ($\beta$ and $\gamma$), red bone marrow (due to its high blood content) ($\beta$ and $\gamma$) and tumour ($\gamma$):

$$D_{rbm} = A_{bld} \times S_{(rbm \leftarrow wb)} + 0.3 \times A_{bld} \times S_{(rbm \leftarrow rbm)} + A_{tum} \times S_{(rbm \leftarrow tum)}$$

where $A_{bld}$ and $A_{tum}$ = as defined above.

$S_{(rbm \leftarrow wb)}$ = $2.90 \times 10^{-6}$ Gy/MBq × hr

0.3 = the proportion of total blood volume estimated to be present in bone marrow.

$S_{(rbm \leftarrow rbm)}$ = $6.20 \times 10^{-5}$ Gy/MBq × h.

and $S_{(rbm \leftarrow tum)}$ = $4.58 \times 10^{-5}$ Gy/MBq × h.

Doses to bone marrow ranged from 0.07–0.51 Gy (mean 0.25). In all cases, the majority (>65%) of the dose to the marrow was as a result of blood circulating activity. As might be predicted from these calculated doses, no myelosuppression was noted in any of the individuals studied. Dose to marrow per MBq of activity injected was less variable than the overall doses, ranging from $5 \times 10^{-5}$–$2.6 \times 10^{-4}$ Gy/MBq (mean $1.5 \times 10^{-4}$). Values for patient 6 are an underestimate as no blood data is available.

The dose to normal brain ($D_{br}$) was calculated as the sum of a 'self-dose' from blood contained within the brain ($\beta$ and $\gamma$ radiation) and a component from the tumours $\gamma$ radiation, using the formula:

$$D_{brain} = \rho \times A_{bld} \times S_{(br \leftarrow br)} + A_{tum}\ S_{(br \leftarrow tum)}.$$

where $\rho$ = Proportion of total blood volume estimated to be present in the brain (33 ml/patient's blood volume).

$S_{(br \leftarrow br)}$ = $1.04 \times 10^{-4}$ Gy/MBq × h.

and $S_{(br \leftarrow tum)}$ = $4.58 \times 10^{-5}$ Gy/MBq × h.

The doses to brain ranged from 2.15–9.23 Gy (mean 5.20); results expected from the prolonged retention of isotope in the tumour cavity. The dose to brain was almost exclusively delivered from $\gamma$ emissions from the tumour site as blood activity only contributed a maximum of 0.017 Gy to the total. No difference in brain dose was apparent whether $^{131}I$ was delivered into either a cystic lesion or tumour resection cavity. Dose per MBq of activity injected ranged from $1.44 \times 10^{-3}$–$5.56 \times 10^{-4}$ Gy/MBq, with a mean of $3.23 \times 10^{-3}$.

Volumes of glioma cysts were calculated from CT scans. In the case of patients having tumour resections, an approximation of the isotope volume of distribution was made from radioimmunoscintigraphic images. In each instance, the tumour was assumed to be spherical. Two estimates of dose to tumour are given, assuming either no antibody binding or 100% binding. This approach was undertaken to show the dose range that can be achieved with this type of therapy as it is difficult to be certain of the degree of antibody binding obtained in each case. The values presented here should, therefore, only be taken as an estimate of the doses that can be achieved via targeting $^{131}I$ by direct injection into a tumour/cystic lesion. For the purpose of this model, only the $\beta$ component of $^{131}I$ was considered. The $\gamma$ component delivered to the tumour will be similar to the whole brain dose as calculated above.

The mean tumour dose to tissue within the $R_{95}$ of $^{131}I$ ($D_{tum}$) is derived both from the activity bound to the periphery and from a shell of $R_{95}$ thickness of unbound activity within the cavity. $R_{95}$ is defined as the thickness of tissue in which 95% of the $\beta$ energy from $^{131}I$ is deposited (0.992 mm).

**Figure 4** Determination of the levels of $^{131}I$ in the blood of patients following intratumoral/intracavity injection of $^{131}I$-ERIC-1. 1 ml blood samples were counted and the level of $^{131}I$ expressed is the % of the injected activity in the blood compartment.

**Table III** Dose to whole body, bone marrow and whole brain following intratumoural/intracavity injection of $^{131}$I-MoAb

| Patient | Whole body dose Total (Gy) | Whole body dose Dose Activity (Gy/MBq) | Bone marrow dose Total (Gy) | Bone marrow dose Dose Activity (Gy/MBq) | Whole brain dose Total (Gy) | Whole brain dose Dose Activity (Gy/MBq) |
|---|---|---|---|---|---|---|
| *Cystic lesions* | | | | | | |
| 1 | 0.15 | $1 \times 10^{-4}$ | 0.16 | $1.1 \times 10^{-4}$ | 4.20 | $2.85 \times 10^{-3}$ |
| 2 | 0.31 | $1.9 \times 10^{-4}$ | 0.25 | $1.5 \times 10^{-4}$ | 9.23 | $5.6 \times 10^{-3}$ |
| *Tumour resections* | | | | | | |
| 3 (a) | 0.87 | $7 \times 10^{-5}$ | 0.07 | $5 \times 10^{-5}$ | 2.60 | $2.0 \times 10^{-3}$ |
| 3 (b) | 0.12 | $9 \times 10^{-5}$ | 0.07 | $6 \times 10^{-5}$ | 3.55 | $2.7 \times 10^{-3}$ |
| 4 | 0.13 | $9 \times 10^{-5}$ | 0.51 | $3.5 \times 10^{-4}$ | 2.15 | $1.4 \times 10^{-3}$ |
| 5 | 0.18 | $1.2 \times 10^{-4}$ | 0.39 | $2.6 \times 10^{-4}$ | 4.22 | $2.8 \times 10^{-3}$ |
| 6[a] | 0.25 | $1.7 \times 10^{-4}$ | 0.06 | $4 \times 10^{-5}$ | 8.03 | $5.1 \times 10^{-3}$ |
| 7 | 0.26 | $1.2 \times 10^{-4}$ | 0.26 | $1.2 \times 10^{-4}$ | 7.62 | $3.5 \times 10^{-3}$ |

[a]Only the component from tumour as no blood data was available.

$$D_{tum} = f \times A_{tum} \times S_{(glioma \leftarrow bound)} + (1\text{-}f) \times F(\text{Volume of source}^{*}/\text{Volume of cavity}) \times A_{tum} \times S_{(glioma \leftarrow unbound)}$$

where $f$ = The fraction of antibody bound to tumour.

$S_{(glioma \leftarrow bound)}^{**}$ = $3.297 \times 10^{-5}$ Gy/MBq × h.

$S_{(glioma \leftarrow unbound)}^{**}$ = $1.039 \times 10^{-5}$ Gy/MBq × h.

*Volume of source depends on the $R_{95}$ of $^{131}$I = 0.992 mm.

**S values quoted here have been calculated in conjuntion with the Department of Medical Physics, Frenchay Hospital.

Assuming a uniform distribution of $^{131}$I within the tumour/cyst cavity (i.e. no antibody binding) estimates of tumour dose ranged from 20 to 164 Gy (mean 51.2) (Table IV). This was calculated to be between six and 25 times the dose received by normal brain. Alternatively, if the ideal situation of 100% antibody binding is achieved, tumour doses increase markedly to between 856 and 4504 Gy (mean 1843). Using these figures, the tumour to normal brain ratio ranged from 187–564:1. It must be remembered, however, that because of the $R_{95}$ of $^{131}$I being relatively short, dose deposition resulting from antibody binding will only affect tumour within 1 mm of the isotope source.

### *Response data*

Both patients with cystic lesions demonstrated a marked reduction in their need for aspiration of cyst fluid. For example, patient 1 needed weekly aspiration prior to therapy, but following infusion of $^{131}$I-ERIC-1 no further aspirations were necessary for a period of 5 months. Patient 1 died 9 months from $^{131}$I-MoAb therapy and patient 2 remains alive with stable disease 10 months after receiving the conjugate. Of the patients with recurrent tumours, patient 3 remained asymptomatic for 5 months, although he underwent debulking surgery to reduce intracranial pressure 2 months after receiving the conjugate. The resected material was extremely necrotic but contained occasional viable tumour cells. He subsequently received a further injection of $^{131}$I-MoAb and is alive with progressive disease 8 months from treatment. Patient 4 had a symptom free period of 5 months before requiring additional therapy, whilst patient 5 remains disease free 5 months from $^{131}$I-MoAb injection. Patients 6 and 7 died 1 and 2 months from treatment. Patient 6 died due to tumour progression distant from the site of MoAb injection.

### Discussion

The infusion of $^{131}$I-MoAbs directly into either a tumour resection cavity or cystic lesion is fundamentally different to using an antibody as a targeting agent. In the former instance, the antibody is used to hold the therapeutic agent in place, rather than to deliver it to malignant cells throughout the body. Our approach can therefore be regarded as a 'liquid phase brachytherapy'.

In this study, we have used a MoAb that recognises NCAM expressed on all gliomas studied ($n > 50$) as well as both normal neural and glial cells (Bourne *et al.*, 1991). Binding is predominantly restricted to tumour, because access to normal brain is limited. This is clearly the case, as scintigraphy after MoAb administration shows that concentration of isotope is confined to the injection site up to 3 weeks after administration of the conjugate (Figure 1). To what degree the radiolabelled MoAb diffuses into normal brain parenchyma adjacent to the tumour is unclear. Very

**Table IV** Dose to tumour assuming either 0 or 100% binding of $^{131}$I-MoAb administered directly into either a tumour cavity or tumour cyst

| Patient | Estimated tumour volume ($cm^3$) | Mean dose to tumour: Assuming 0% binding Total (Gy) | Assuming 0% binding Dose Activity (Gy/MBq) | Assuming 100% binding Total (Gy) | Assuming 100% binding Dose Activity (Gy/MBq) |
|---|---|---|---|---|---|
| *Cystic lesions* | | | | | |
| 1 | 58 | 27 | 0.018 | 1451 | 0.98 |
| 2 | 94 | 36 | 0.022 | 1733 | 1.06 |
| *Tumour resections* | | | | | |
| 3 (a) | 47 | 20 | 0.015 | 1225 | 0.92 |
| 3 (b) | 46 | 28 | 0.021 | 1071 | 0.79 |
| 4 | 30 | 26 | 0.017 | 856 | 0.57 |
| 5 | 46 | 34 | 0.022 | 1270 | 0.84 |
| 6 | 18 | 164 | 0.10 | 4504 | 2.87 |
| 7 | 38 | 75 | 0.034 | 2635 | 1.20 |

preliminary observations using SPECT indicate that diffusion is limited to 0.5–1.0 cm from the site of infusion, although this needs further study. A small degree of diffusion is important if this approach to therapy is to work, as it is clear that whilst glioma cells usually do not grossly metastasise, they can invade normal brain parenchyma adjacent to the tumour edge.

The selection of a MoAb that cross reacts with tumour and normal tissue was deliberate, as this should limit diffusion to the immediate vicinity of the injection site. Diffusion should be restricted by binding to a gross excess of antigen expressed on normal brain infiltrated by or surrounding the tumour. Furthermore, this should create a localised area bathed in $^{131}I$ exposing any invading malignant cells to a 'crossfire' effect from the $\beta$-emissions from the $^{131}I$ attached to the MoAb binding to normal neural elements. If a 'tumour specific' MoAb is used that only binds to a limited number of malignant glial cells in the normal brain parenchyma, theoretically neither of these effects will occur.

The difference between instilling $^{131}I$-MoAbs into tumour cavities/cysts and the use of isotope colloid is that a degree of targeting can be obtained through the use of a MoAb. Even accepting the potential errors that occur in the calculation of dose to tumour, it is clear that a major therapeutic advantage can be conferred by binding $^{131}I$ to tumour and adjacent brain. This is predominantly due to the short path length of $^{131}I$ with an $R_{95}$ of 0.992 mm. However, this also implies that the mean doses reported for targeting in Table IV are only going to be delivered to a rim of tumour approximately 1.0 mm thick. With $^{131}I$ as the isotope, diffusion becomes critical so that the volume of tumour exposed to $\beta$ radiation is increased.

It is clear from the nature of glioma development, that the choice of $^{131}I$ for conjugation to the MoAb is far from ideal. This isotope is a relatively short range $\beta$ emitter that also has a $\gamma$ component. A higher energy pure $\beta$ emitter such as $^{90}Y$ is theoretically a much better candidate for use in this situation (Moi *et al.*, 1990). We have refrained from the use of $^{90}Y$ because of concerns regarding myelosuppression seen in other studies (Stewart *et al.*, 1990; Vriesendorp *et al.*, 1991). To overcome this problem, we initiated laboratory studies on the conjugation of $^{90}Y$ to MoAb, through the use of macrocycles. However, in the light of the pharmacokinetics revealed in this study, we believe a more simplistic approach involving DTPA-MoAb conjugates as the carrier for $^{90}Y$ should be feasible. Blood levels of isotope have been very low and, as a consequence, if $^{90}Y$-MoAb conjugates behave in a similar fashion to $^{131}I$ MoAbs, there should be little concern with respect to damage to bone marrow. An alternative isotope of interest for targeting could be $^{32}P$, another $\beta$ emitter with a longer half-life than $^{90}Y$. Difficulties in the conjugation of $^{32}P$ to MoAbs have recently been resolved, although the stability of such constructs requires further investigation.

With respect to $^{131}I$-MoAbs, myelosuppression is clearly not going to be the primary toxicity of this approach to therapy. Administration of single doses of $^{131}I$ to patients will be limited by concerns regarding exposure of nursing and medical staff to patients with high levels of $^{131}I$ in their tumours. For example, measurement of tumour dose by an external probe resulted in staff receiving 4 $\mu$Sv per exposure to the patient and this was the reason for changing our method of determining tumour clearance. In this respect, the use of alternative radionuclides such as $^{90}Y$ is also greatly preferable.

Peak blood levels of $^{131}I$-MoAbs were clearly higher in two patients (4 and 5) as compared to the other five. These individuals received their tracer and therapy studies at different times from surgery. Clearance from a tumour cavity does not, therefore, depend just on the area sealing rapidly after surgery. It is clear from the two MoAb administrations to patient 3 that the patency of the tumour cavity is critical to obtain retention of the antibody at the injection site. To check this, all patients receive a tracer study with $^{131}I$-MoAb prior to therapy and we are now waiting a minimum of 3 weeks from surgery before instilling MoAbs into tumour resection cavities.

Estimates of tumour dosimetry are dependent upon an accurate determination of tumour volume and the degree of MoAb binding occurring in each patient. Both of these values are extremely difficult to measure and are prone to considerable error. Whilst estimation of the volume of tumour cysts can be made from CT scans in patients having debulking surgery, the best estimate of cavity volume can be made from nuclear medicine images. Here, one is determining the volume of isotope distribution which includes any areas of localised diffusion that have taken place. Furthermore, to enable the calculation of dose, the tumour shapes are assumed to be spherical, which is an obvious oversimplification. The degree of MoAb binding will also vary from patient to patient and will change with respect to the time from injection. In four of the eight cases studied, we have withdrawn small volumes of fluid from the Ommaya reservoir 8 to 19 days after injecting the $^{131}I$-MoAb conjugate. Counting this material and extrapolating to the whole volume of distribution revealed a range of binding of 8–80% of the injected dose. Using the dosimetry model above, tumour doses of 110, 238, 236 and 3768 Gy for patients 1, 2, 3 (second injection) and 7 respectively would occur. Interestingly, in each case studied, $^{131}I$ was largely attached to the MoAb which also remained immunoreactive in *in vitro* assays.

Rather than continuing escalating the dose of $^{131}I$-MoAb given as a single injection, we are proposing to investigate repeated therapy in patients with either tumour resection cavities or cystic lesions. It is clear from our studies on the intraventricular injection of MoAbs to patients with diffuse leptomeningeal spread of tumour, that repeated injections of mouse MoAbs will result in the generation of a systemic anti-mouse Ig response. How this will affect the clearance of MoAb from a cystic lesion or tumour cavity is not clear. No effect was noted in patient 3, as clearance from the tumour after the second injection was considerably longer than the first. However, no systemic anti-mouse Ig response was noted in this patient.

Following repeated intrathecal injections of $^{131}I$-MoAbs, we have demonstrated that the anti-mouse Ig observed systemically is not mirrored, to the same extent, within the intrathecal compartment. This may also be the case for intratumoral lesions. Alternatively, if the dosimetry presented here is correct, the initial injection of $^{131}I$-MoAb will certainly cause tumour necrosis. This, in turn, might make the area more 'leaky' and as a result enhance the loss of conjugate from the injection site. Furthermore, the injection of MoAbs into the tumour may cause an invasion of macrophages. Such cells, through phagocytosis, could adversely affect the residence time of MoAbs in the tumour site.

The purpose of this investigation was to undertake a pilot study on the practicability of injecting radiolabelled MoAbs into tumour cysts/cavities. Bearing in mind the aggressive nature of the tumours being treated, it is unreasonable to expect to see prolonged tumour responses from a single injection of $^{131}I$-MoAb. However, it is clear from the two patients with cystic lesions that responses to $^{131}I$-MoAbs do occur. To obtain accurate response data on patients having $^{131}I$-MoAbs after debulking surgery for relapsed disease would require a study randomising patients to either receiving or not receiving antibody instillation. This, we believe, is not the correct approach at the current time. More important, is to establish the principles involved in repeat therapy and explore the use of $^{90}Y$-MoAbs in this context. If results from these preliminary studies are promising, we believe it justifiable to initiate a study incorporating $^{90}Y$-MoAb therapy into the primary treatment of patients with glioma. Our projected plan would be to treat all patients with conventional surgery and radiotherapy and subsequently randomise the group to receive either $^{90}Y$-MoAb or no therapy. Whilst this is a long way from the original concept of using MoAbs as targeting agents, it may prove to be one of the ways in which they can be exploited most successfully.

We would like to thank the Imperial Cancer Research Fund for their support and funding of this project. We would also like to thank the medical, nursing and radiology staff at both the Bristol Royal Infirmary and Frenchay Hospital for their assistance. We also thank Sharon Standen for typing this manuscript.

### References

BOURNE, S.P., PATEL, K., WALSH, F., POPHAM, C.J., COAKHAM, H.B. & KEMSHEAD, J.T. (1991). A monoclonal antibody (ERIC-1), raised against retinoblastoma, that recognises the neural cell adhesion molecule (NCAM) expressed on brain and tumours arising from the neuroectoderm. *J. Neuro-Oncology*, **10**, 111–119.

EPENETOS, A.A., MUNRO, A.J., STEWART, S., RAMPLING, R., LAMBERT, H.E., MCKENZIE, L.G., SOUTTER, P., RAHEMTULLA, A., HOOKER, G., SIVLLAPENKO, G.B., SNOOK, S.J., COURTENAY-LUCK, N., HOKIA, D., CRAUZE, T., TAYLOR-PAPADIMITTRIOU, J., DURBIN, A. & BODMER, W. (1987). Antibody-guided irradiation of advanced ovarian carcinoma with intra-peritoneally administered radiolabelled monoclonal antibodies. *J. Clin. Oncol.*, **12**, 1890–1899.

FRAKER, P.J. & SPECK, J.C. (1978). Protein and cell membrane iodinations with a sparingly soluble chloroamide, 1,3,4,6-Tetrachloro-3a,6a-diphenylglycoluril. *Biochem. & Biophys. Res. Comms.*, **80**, 849–857.

HALPERIN, E.C., BURGER, P.C. & BULLARD, D.E. (1988). The fallacy of the localised supratentorial malignant glioma. *Int. J. Radiat. Oncol. Biol. Phys.*, **15**, 505–509.

JAIN, R.K. (1990). Vascular and interstitial barriers to delivery of therapeutic agents in tumors. *Cancer Metastasis Rev.*, **9**, 253–266.

JAIN, R.K. (1991). Haemodynamic and transport barriers to the treatment of solid tumours. *Int. J. Radiat. Biol.*, **60**, 85–100.

KEMSHEAD, J.T., JONES, D.H., GOLDMAN, A., RICHARDSON, R.B. & COAKHAM, H.B. (1984). Is there a role for radioimmunolocalisation in the diagnosis of intracranial malignancies? *J. Roy. Soc. Med.*, **77**, 847–854.

LASHFORD, L.S., DAVIES, A.G., RICHARDSON, R.B., BOURNE, S.P., BULLIMORE, J.A., ECKERT, H., KEMSHEAD, J.T. & COAKHAM, H.B. (1988). A pilot study of $^{131}$I-monoclonal antibodies in the therapy of leptomeningeal tumours. *Cancer*, **61**, 857–868.

LINDMO, T., BOVEN, E., CUTTITTA, F., FEDORKO, J. & BUNN, P.A. (1984). Determination of the immunoreactive fraction of radiolabelled monoclonal antibodies by linear extrapolation to binding at infinite antigen excess. *J. Immunol. Methods*, **72**, 77–89.

MEDICAL INTERNATIONAL RADIATION DOSE COMMITTEE (MIRD). (1971). *J. Nucl. Med.*, **12**, 1–32.

MOSELEY, R.P., DAVIES, A.G., RICHARDSON, R.B., ZALUTSKY, M., CARRELL, S., FABRE, J., SLACK, N., BULLIMORE, J., PIZER, B., PAPANASTASSIOU, V., KEMSHEAD, J.T., COAKHAM, H.B. & LASHFORD, L.S. (1990). Intrathecal administration of $^{131}$I-monoclonal antibody as a treatment for neoplastic meningitis. *Br. J. Cancer*, **62**, 637–642.

MOI, M.K., DENARDO, S.J. & MEARES, C.F. (1990). Stable bifunctional chelates of metals used in radiotherapy. *Cancer Res.*, **50**, (Suppl. 3) 789s–793s.

PIZER, B., PAPANASTASSIOU, V., HANCOCK, J., CASSANO, W., COAKHAM, H.B. & KEMSHEAD, J.T. (1991). A pilot study of monoclonal targeted radiotherapy in the treatment of central nervous system leukaemia in children. *Br. J. Haem.*, **77**, 466–472.

SANDS, H., JONES, P.L., SHAH, S.A., PALME, D., VESSELLA, R.L. & GALLAGHER, B.M. (1988). Correlation of vascular permeability and blood flow with monoclonal antibody uptake by human colon and renal cell xenografts. *Cancer Res.*, **48**, 188–193.

STEWARD, D.J. (1989). The role of chemotherapy in the treatment of gliomas in adults. *Cancer Treat. Revs.*, **16**, 129–160.

STEWART, J.S., HIRD, V., SNOOK, D., DHOKIA, B., SIVOLAPENKO, G., HOOKER, G., PAPADIMITRIOU, J.T., ROWLINSON, G., SULLIVAN, M. & LAMBERT, H.E. (1990). Intraperitoneal $^{90}$Y-monoclonal antibody in ovarian cancer. *J. Clin. Oncol.*, **8**, 1941–1950.

VRIESENDORP, H.M., HERPST, J.M., GERMACK, M.A., KLEIN, J.L., LEICHNER, P.K., LOUDENSLAGER, D.M. & ORDER, S.E. (1991). Phase I-II studies of yttrium-labelled anti-ferritin treatment for end-stage Hodgkin's disease, including Radiation Therapy Oncology Group 87-01. *J. Clin. Oncol.*, **9**, 918–928.